# Overview of Ferroptosis and Synthetic Lethality Strategies

**DOI:** 10.3390/ijms22179271

**Published:** 2021-08-27

**Authors:** Yuko Kinowaki, Towako Taguchi, Iichiroh Onishi, Susumu Kirimura, Masanobu Kitagawa, Kouhei Yamamoto

**Affiliations:** 1 Department of Comprehensive Pathology, Graduate School of Medical and Dental Sciences, Tokyo Medical and Dental University, 1-5-45 Yushima, Bunkyo-ku, Tokyo 113-8519, Japan; taguchi.pth2@tmd.ac.jp (T.T.); masa.pth2@tmd.ac.jp (M.K.); 2 Division of Surgical Pathology, Tokyo Medical and Dental University Hospital, 1-5-45 Yushima, Bunkyo-ku, Tokyo 113-8519, Japan; iichpth2@tmd.ac.jp (I.O.); kirimura.pth2@tmd.ac.jp (S.K.)

**Keywords:** ferroptosis, synthetic lethality, CRISPR-Cas9 screen, GPX4, FSP1

## Abstract

Ferroptosis, a term first proposed in 2012, is iron-dependent, non-apoptotic regulatory cell death induced by erastin. Ferroptosis was originally discovered during synthetic lethal screening for drugs sensitive to RAS mutant cells, and is closely related to synthetic lethality. Ferroptosis sensitizes cancer stem cells and tumors that undergo epithelial−mesenchymal transition and are resistant to anticancer drugs or targeted therapy. Therefore, ferroptosis-inducing molecules are attractive new research targets. In contrast, synthetic lethal strategies approach mechanisms and genetic abnormalities that cannot be directly targeted by conventional therapeutic strategies, such as RAS mutations, hypoxia, and abnormalities in the metabolic environment. They also target the environment and conditions specific to malignant cells, have a low toxicity to normal cells, and can be used in combination with known drugs to produce new ones. However, the concept of synthetic lethality has not been widely adopted with ferroptosis. In this review, we surveyed the literature on ferroptosis-related factors and synthetic lethality to examine the potential therapeutic targets in ferroptosis-related molecules, focusing on factors related to synthetic lethality, discovery methods, clinical application stages, and issues in drug discovery.

## 1. Introduction

Apoptosis, originally termed by Kerr and Wyllie in 1972 [1], is regulated cell death (RCD), a process that is distinct from accidental cell death (ACD), and its molecular mechanisms have been elucidated. The results of this research have led to an understanding of physiological biological mechanisms, the nature of cancer, and applications as therapeutic strategies against cancer [2,3]. Subsequently, necrosis, which was initially thought to be ACD, was also found to have a molecular mechanism similar to RCD, indicating that RCD is not limited to apoptosis [4]. In 2003, the small-molecule erastin was discovered to selectively induce cell death in genetically engineered cells with oncogenic RAS mutations, and in 2012, a previously unknown type of RCD characterized as iron-dependent, nonapoptotic cell death induced by erastin was termed ferroptosis [5]. Ferroptosis is cell death caused by cell membrane damage due to lipid peroxidation, accompanied by the iron-dependent production of reactive oxygen species (ROS) [5]. Many unknown mechanisms of ferroptosis remain, and further research is needed to determine its regulators and whether it is a truly independent type of RCD.

In contrast, synthetic lethality (also known as synthetic lethal) is a phenomenon in which mutations in either of two genes have no effect on cell survival, but abnormalities in both genes lead to cell death [6]. Synthetic lethality is observed in loss-of-function mutations (as in tumor suppressor genes), but it can also be found in gain-of-function mutations (as in oncogenes). Synthetic lethal cytotoxicity caused by certain intrinsic conditions, such as genetic background, hypoxia, or metabolic changes, or extrinsic conditions like treatment with DNA-damaging agents, is referred to as conditional synthetic lethality [7].

To date, more than 90 targeted cancer drugs have been developed [7,8], most of which are small molecules or antibodies that target gain-of-function mutations in oncogenes. In contrast, it is difficult to restore the function of proteins encoded by inactivated tumor suppressor genes, hampering the development of anticancer drugs targeting loss-of-function mutations in tumor suppressor genes [7,8]. However, it is anticipated that the mechanism of synthetic lethality can be used to overcome this barrier, thereby providing a basis for the identification of genes that exhibit synthetic lethality with tumor suppressor genes.

Ferroptosis is defined as “cell death due to synthetic lethality caused by increased RAS activity and ROS” [9]; it was discovered in the screening of drugs sensitive to RAS mutant cells and is closely related to synthetic lethality. Ferroptosis-related molecules are new targets of research, but they have attracted much attention because of their unique properties, which are described in this review. Although both ferroptosis and synthetic lethality are relatively new concepts, ferroptosis is cell death that is closely related to the cellular environment, such as oxidative stress and metabolic abnormalities, and is highly compatible with synthetic lethal strategies, all of which are appealing in that new drug effects can be expected by pairing with existing drugs.

In this review, we introduce several reports related to ferroptosis regulators and synthetic lethality, and categorize the literature based on the presence or absence of RAS mutations. For each study, we focused on the factors related to synthetic lethality, the specific search method, the stage of clinical application, and issues in drug discovery, as well as examined potential therapeutic targets in ferroptosis-related molecules.

## 2. Overview of Ferroptosis

Cell death can be divided into ACD and RCD. RCD has a tightly structured signaling cascade and molecularly defined effector mechanisms, the most common of which is apoptosis [4]. In 2003, pioneering research in the study of ferroptosis found that the small-molecule erastin selectively induced cell death in genetically engineered cells with oncogenic RAS mutations [10], and in 2012, the term ferroptosis was coined for a previously unknown type of RCD that was erastin-induced and iron-dependent nonapoptotic cell death [5]. Ferroptosis is a unique iron-dependent form of programmed cell death driven by lipid peroxidation in cells [5], and it is distinct from necrosis, apoptosis, and autophagy [4,5,11,12]. Morphologically, the characteristics of ferroptosis-induced cell death are shrinkage of the mitochondria with increased membrane density and a reduction in or the disappearance of mitochondrial cristae. Furthermore, ferroptosis does not have the morphological features of typical apoptosis (e.g., chromatin condensation and margination), necrosis (e.g., cytoplasmic and organelle swelling, and plasma membrane rupture), and autophagy (e.g., formation of double membrane-enclosed vesicles) [5,11,12,13,14] (Table 1). Despite this understanding, many unknown mechanisms for ferroptosis remain, and further research is warranted to determine its regulators and whether it is a truly independent type of RCD.

**Figure 1:**
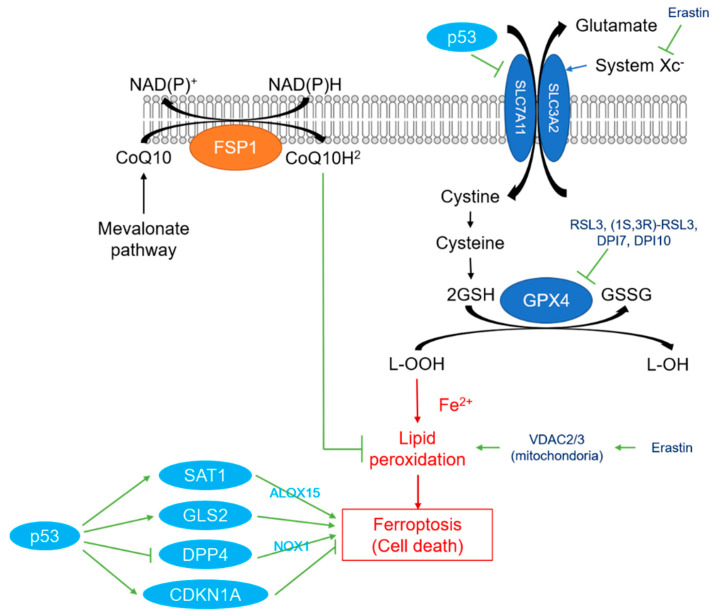
Schematic diagram of the ferroptosis signaling pathway. The ferroptosis pathway is triggered by several different classes of small molecules, centered on GPX4 and FSP1. Glutathione peroxidase 4 (GPX4) hydrolyzes lipid peroxides to harmless lipid alcohols (-OH). GPX4 requires glutathione (GSH) as a cofactor, which is oxidized by GPX4 (GSSG) and then reduced to GSH by glutathione reductase (GR). GSH synthesis is dependent on cysteine transported by system Xc- (also called SLC7A11). Ferroptosis suppressor protein 1 (FSP1) produces ubiquinol from ubiquinone independently of GSH, and acts as a lipophilic radical scavenger in the membrane, protecting it from ferroptosis. Oxidative phosphorylation (OXPHOS) and tricarboxylic acid (TCA) cycles are required for ferroptosis caused by cystine depletion and system Xc-. p53 acts positively against ferroptosis by promoting SAT1, GLS2, and CDKN1A, and inhibiting DPP4 and SLCA11. It is thought that p53 acts both positively and negatively on ferroptosis.

Previous studies have reported that ferroptosis is regulated by multiple genes, but the major regulators are glutathione peroxidase 4 (GPX4) and ferroptosis suppressor protein 1 (FSP1) [15,16,17] (Table 2 and Figure 1).

GPX4 is a member of the GPX family, consisting of GPX1 to GPX8. It converts the small peptide glutathione (GSH) to oxidized glutathione and reduces cytotoxic lipid peroxide (L-OOH) to the corresponding alcohol (L-OH) [15,16]. GPX4 also has an antioxidant effect on cell membrane damage and lipid peroxidation in ferroptosis. The inhibition of GPX4 causes lipid peroxidation accumulation and the induction of ferroptosis [13]. The ferroptosis inducer (1S,3R)-RSL3 (RSL3) and the compounds DPI7 and DPI10 directly affect GPX4 and inhibit its activity, resulting in ferroptosis [15]. GSH synthesis, which is affected by GPX4, involves an amino acid antitransporter called System Xc-, which is widely distributed in phospholipids [5]. System Xc- is a heterodimer composed of two subunits, SLC7A11 and SLC3A2. Glutamine and cystine are exchanged at a ratio of 1:1, and cystine is taken up into cells where it is reduced to the GSH precursor cysteine. Cysteine affects GSH because GSH is a tripeptide consisting of glutamic acid, cysteine, and glycine [15]. Therefore, system Xc- inhibition results in the inhibition of the intracellular uptake of cystine, GSH reduction, decreased GPX4 activity, and lipid ROS accumulation, causing oxidative damage and ferroptosis. Furthermore, it was reported that p53 downregulates the expression of SLC7A11 in heterodimers of system Xc-, thereby inhibiting cystine uptake by system Xc-, and consequently, the induction of ferroptosis [18,19]. The role of p53 is controversial as it both promotes and inhibits ferroptosis. The activation of spermidine/spermine N1-acetyltransferase 1 (SAT1), a transcriptional target gene for p53, induces ROS production, lipid peroxidation, and ferroptosis, and correlates with expression levels of arachidonate 15-lipoxygenase (ALOX15) [20]. Additionally, glutaminase 2 (GLS2) has been identified as a transcriptional target for p53 [21], and that knockout of GLS2 inhibits ferroptosis [22]. Although there are reports that p53 enhances ferroptosis, several studies have reported that p53 suppresses ferroptosis. In colorectal cancer (CRC), the depletion of p53 prevents the nuclear accumulation of dipeptidyl peptidase-4 (DPP4), and consequently, binding to the membrane-related DPP4-mediated trigger NADPH oxidase 1 (NOX1), thereby inducing lipid peroxidation and ferroptosis [23]. Furthermore, p53-mediated expression of cyclin-dependent kinase inhibitor 1A (CDKN1A, also known as p21) results in resistance to ferroptosis [24]. p53 is an important tumor suppressor gene involved in many critical cellular processes, such as cell cycle arrest, apoptosis, and metabolism [25]; however, its role in ferroptosis is unclear and warrants further investigation.

In 2019, a gene encoding a protein named FSP1 (previously called apoptosis-inducing factor mitochondrial 2 (AIFM2)) was identified as a ferroptosis suppressor that functions independently of GPX4 in the plasma membrane [26,27,28]. FSP1 functions as an NADPH-dependent CoQ oxidoreductase and reduces CoQ10 (also known as ubiquinone-10), which is a product of the mevalonate pathway. The reduced CoQ10 then acts as a lipophilic radical-trapping antioxidant that prevents lipid peroxidation and ferroptosis. It was also revealed that the translocation of FSP1 to the plasma membrane requires the N-myristoylation of FSP1. Thus, the NADPH-FSP1-CoQ10 pathway is a strong inhibitor of lipid peroxidation and ferroptosis. Additionally, among many cultured human cancer cell lines, the level of resistance to ferroptosis is positively correlated with the FSP1 expression level, suggesting that changes to FSP1 are clinically significant. Furthermore, in 2021, dihydroorotate dehydrogenase (DHODH) was discovered, and it is a regulator of mitochondrial membrane ferroptosis [29].

## 3. Overview of Synthetic Lethality

Synthetic lethality is classically defined as a setting in which the inactivation of either of two genes individually has little effect on cell viability, but the loss of function of both genes simultaneously leads to cell death [6,30]. In a broader context, synthetic lethality includes cases where the presence of two mutations is more detrimental to cell survival than of either mutation alone (also known as synthetic sickness) [7]. Synthetic lethality is most often found in loss-of-function mutations like in cancer suppressor genes, but is also found in gain-of-function mutations like in oncogenes. The main scenarios of synthetic lethality are as follows [31]: (1) loss-of-function mutations in gene A alone are viable, but mutations in gene B cause cell death; (2) loss-of-function mutations in gene A alone are viable, but inhibition of gene B leads to cell death; (3) overexpression of gene A alone is viable, but inhibition of gene B causes cell death; and (4) synthetic cytotoxicity occurs due to certain intrinsic conditions, such as genetic background, hypoxia, and metabolic changes, or extrinsic conditions like treatment with DNA-damaging agents. The latter is referred to as conditional synthetic lethality [31].

For example, PARP inhibitors used to treat hereditary breast and ovarian cancer syndrome correspond to synthetic lethality scenario (2) [7,8], but synthetic lethality associated with ferroptosis varies and can be any of the four.

## 4. Anticancer Drug Discovery Using Synthetic Lethality

More than 90 targeted cancer therapies have been developed for nearly 30 types of cancer [32], most of which are small molecules or antibodies that target gain-of-function mutations in oncogenes. However, in contrast, it remains difficult to restore the function of proteins encoded by inactivated tumor suppressor genes, as strategies that directly target loss-of-function mutations must target misfolded or partially missing proteins or proteins that are expressed or knocked out [33]. Such a challenge hampers the development of anticancer drugs targeting loss-of-function mutations in tumor suppressor genes [31]. However, there is optimism that the mechanism of synthetic lethality can be used to overcome this barrier, providing a basis for the identification of genes that exert synthetic lethality with tumor suppressor genes. Recently, the combination of BRCA1/2 mutations observed in breast and ovarian cancer and PARP inhibitors has attracted attention as an example of the clinical application of drugs using the principle of synthetic lethality [8,34].

Synthetic lethality has been studied in yeast for years because of the ease of genetic modification [31]. The identification of proteins homologous to yeast in humans, particularly those involved in DNA damage and repair, has led to attempts to use synthetic lethality in cancer treatment [30]. Furthermore, with the elucidation of the entire genetic sequence of the human genome, it is possible to search for synthetic lethal agents using RNA interference (RNAi) screening methods. Recently, the emergence of CRISPR-based tools and the diversification of methods to facilitate functional genomics have greatly increased the speed and robustness of synthetic lethal target discovery [30]. Using these techniques, researchers have analyzed the context-specific genetic dependencies identified in genomic screens for loss-of-function cancer cell lines, and found that synthetic lethal interactions are abundant [35,36].

The results of these analyses are provided to the Cancer Dependency Map (DepMap) [37], which is a collaborative project that is building a comprehensive database of new drug targets and biomarker candidates through projects such as Project Achilles [38], Project DRIVE (Novartis) [35], and Project Score (Sanger Institute) [39,40], all of which are also contributing data to DepMap [37]. Importantly, the resources generated using this large-scale targeted discovery approach have enabled the characterization of genetic interaction networks and the identification of synthetic lethal cancer targets with a potential drug efficacy [41,42].

## 5. Synthetic Lethality and Ferroptosis

A definition of ferroptosis is “cell death by synthetic lethality due to increased RAS activity and ROS”; it was originally discovered during the screening of drugs sensitive to RAS mutant cells [9]. Ferroptosis-related substances are new targets of research and have attracted much attention because of several properties, such as (i) susceptibility in sarcomas and tumors after epithelial−mesenchymal transition, which are resistant to conventional anticancer drugs and molecular targeted drugs [43]; (ii) susceptibility to cancer stem cells [44]; and (iii) CD8+ T cells activated by immune checkpoint inhibitors that induce ferroptosis in some types of cancer cells [45]. In this review, we introduce and discuss reports of synthetic lethal studies on ferroptosis-related substances reported to date.

Among ferroptosis-related substances, we targeted factors that positively regulate ferroptosis, such as TFR1, ACSL4, NCOA4, and VDAC2/3, and factors that negatively regulate ferroptosis, such as GPX4, SLC7A11, FSP1, NRF2 (also known as NFE2L2), HSPB1, and HSPA5. A PubMed search using these keywords, plus “Ferroptosis,” yielded 36 results (retrieved on 22 May 2021). Of these 36 articles, we excluded three review articles and 14 articles on the basis that the terms were only briefly mentioned in the preamble or discussion, resulting in the inclusion of 19 articles in this review. Focusing on the genes related to synthetic lethality, specific search method, clinical application stage, and problems in drug discovery in each study, we examined ferroptosis-related molecules as potential therapeutic targets. The approval status of the drugs can be found in the KEGG DRUG database (accessed on 22 May 2021. https://www.genome.jp/kegg/drug/).

## 6. Synthetic Lethality and Ferroptosis—Related to RAS Mutation

Several studies focusing on RAS mutant cells marked the beginning of the discovery of ferroptosis [13,46,47,48,49,50] (Table 3). Although gain-of-function mutations in RAS occur in RAS mutant cells, it is unproven whether they directly result in synthetic lethality, as described in the synthetic lethality scenario (3), and the genetic or metabolic abnormalities that occur in RAS mutant cells are unknown. However, in the cellular environment caused by gain-of-function mutations in RAS or in a state of dependence on a specific gene, if pharmacological or genetic mutation manipulation causes ferroptosis, it falls under the four synthetic lethality scenarios described above [30,33].

The research group of Yang et al., considered pioneers in ferroptosis research, attempted to identify common central regulators for lethality of the ferroptosis inducers RSL3 [13], ML162, and DP110 [63], as well as other small molecules identified using high-throughput synthetic lethal-screening methods against immortalized cells of mutant RAS (HRAS G12V)-expressing BJ fibroblasts. They attempted to identify a common central regulator of the lethality of ferroptosis-inducing small molecules [13,15]. Using standardized metabolomics profiling, they identified a group of compounds that cause glutathione depletion and discovered that these compounds inactivate members of the GPX family. Based on findings from the GPX4 overexpression and knockdown experiments, they identified 12 ferroptosis-inducing factors and confirmed they are different from other RCDs in cell death. Additionally, two major ferroptosis-inducing factors, erastin and RSL3, prevented tumor growth in a xenograft mouse tumor model, and sensitivity profiling of 117 cancer cell lines showed that diffuse large B-cell lymphoma and renal cell carcinoma were highly sensitive to GPX4-modulated ferroptosis. They also found that cancer cell lines with RAS mutations were not selectively lethal to erastin-induced ferroptosis in RAS wild-type cell lines, indicating that although RAS mutations increase susceptibility to ferroptosis, other factors are involved in ferroptosis susceptibility.

Chio et al. [46] showed that NRF2 is required for the maintenance of pancreatic cancer growth by regulating mRNA translation, based on the finding that mutant KRAS causes Nrf2, a key regulator of redox, to induce pancreatic and lung carcinogenesis [64]. Furthermore, they found that NRF2 deficiency resulted in defective autocrine epidermal growth factor receptor (EGFR) signaling and oxidation of specific translational regulatory proteins, thereby leading to impaired cap-dependent and cap-independent mRNA translation in pancreatic cancer cells. Treatment with both MK2206 and L-buthionine-(S, R)-sulfoximine (BSO), which inhibit the EGFR effector AKT and glutathione synthase, respectively, mimicked the NRF2 depletion state and potently inhibited pancreatic cancer growth in KRAS- and TP53-mutated Suit2 PDA cell line and mouse models. Although these findings reveal a promising synthetic lethal strategy for disease treatment, it is not known whether the observed synthetic lethality is ferroptosis.

In contrast, Kwon et al. also elucidated the activity of transcription factors such as NRF2 and AhR, which is a molecular biomarker of erastin-dependent ferroptosis, in a human lung cancer cell model. They constructed a nuclear receptor metapathway (NRM) model, integrating the gene expression of the NRM, and announced that this pharmacogenomic approach predicts erastin sensitivity even in unknown cell lines [48].

Hu et al. [47] performed metabolomic analysis to elucidate the metabolic vulnerability of KRAS-mutated lung adenocarcinoma to treatment and reported that the SLC7A11/glutathione pathway exhibits oncogenic KRAS and metabolic synthetic lethality. Their analysis revealed that when KRAS is activated by mutations, intracellular cystine levels and glutathione biosynthesis are markedly increased. In addition, SLC7A11, a cystine/glutamate antiporter that specifically uptakes cystine, was overexpressed in patients with KRAS-mutated lung adenocarcinoma and positively associated with tumor progression. Furthermore, genetic deletion of SLC7A11 or pharmacological inhibition with sulfasalazine (SAS) selectively killed KRAS mutant cancer cells in vitro and inhibited tumor growth in vivo, suggesting the functionality and specificity of SLC7A11 as a therapeutic target. They also screened the inhibitory effects of certain compounds on glutathione production in 549 cell types, and found that a series of chemicals with a benzotriazole skeleton caused a marked decrease in glutathione production, from which they identified the potent SLC7A11 inhibitor HG106. Specifically, they discovered that HG106 markedly reduced cystine uptake and intracellular glutathione biosynthesis, exhibited selective cytotoxicity against KRAS mutant cells, and increased oxidative and endoplasmic reticulum stress-mediated cell apoptosis (mitochondrial swelling was confirmed by transmission electron microscopy). Although this SLC7A11 inhibitor is a ferroptosis inducer, its effect on autophagy and ferroptosis was concluded to be negative based on LC3 protein measurement, which is an indicator of autophagy, and iron chelator deferoxamine administration experiments. Furthermore, the treatment of KRAS-mutated lung adenocarcinoma with HG106 in several preclinical lung cancer mouse models resulted in marked tumor suppression and prolonged survival. These results indicate that KRAS mutant lung adenocarcinoma cells are vulnerable to SLC7A11 inhibition; however, as HG106 is not an approved drug from the United States Food and Drug Administration (FDA), efforts shifted toward SAS as it is FDA approved.

Sugiyama et al. [49] investigated the effect of the xCT inhibitor SAS on the cytotoxicity of paclitaxel-sensitive and -resistant uterine serous carcinoma cell lines. The increased production of ROS and the activation of the c-Jun N-terminal kinase (JNK) pathway, a downstream target of the RAS signaling pathway, in paclitaxel-resistant cells indicated that the synthetic lethal interaction between ROS accumulation and RAS effector JNK pathway activation is important for enhancing susceptibility to the xCT inhibitor SAS. In turn, SAS is important for enhancing susceptibility to xCT-mediated ferroptosis. In this study, immunoblotting analysis and cell death assay using a ferroptosis inhibitor showed that SAS-induced cell death is not apoptosis but ferroptosis.

## 7. Synthetic Lethality and Ferroptosis—Unrelated to RAS Mutation

In addition to RAS mutations, synthetic lethality is caused by several other factors related to changes in the genes involved in intracellular oxidative stress and the metabolic environment (Table 3).

### 7.1. Related to Ferroptosis Inhibitors

The discovery of the major ferroptosis regulator FSP1 by the independent research groups of Bersuker and Doll in 2019 is also closely related to synthetic lethal screening [27,28]. The existence of cancer cell lines resistant to the GPX4 inhibitor RSL3 prompted Doll et al. [27] and Bersuker et al. [28] to explore alternative mechanisms to prevent ferroptosis, concurrently discovering that FSP1 protects human cells from ferroptosis [65].

Doll et al. generated a cDNA expression library from a GPX4 knockout human breast cancer cell-derived MCF7 cell line and screened for genes complementing GPX4 deficiency, from which they identified FSP1. To validate FSP1 function, they confirmed that the stable expression of FSP1 in mouse Pfa119 and human fibrosarcoma HT1080 cells strongly protected them from pharmacological and genetic inducers of ferroptosis and allowed indefinite proliferation, thus avoiding synthetic lethality. Furthermore, it was determined that the antiferroptotic activity of FSP1 was independent of intracellular glutathione levels, GPX4 activity, ACSL4 expression, and oxidizable fatty acid content, revealing a GPX4-independent mechanism of FSP1-mediated suppression of ferroptosis [66].

Bersuker et al. [28] performed a synthetic lethal CRISPR-Cas9 screen in a ferroptosis-resistant cell line and found that FSP1 protected human cells from ferroptosis. To identify ferroptosis-resistant genes, they used a sublibrary of single guide RNAs (sgRNAs) targeting genes associated with apoptosis and cancer in human U2OS osteosarcoma cells treated with the GPX4 inhibitor RSL3 for synthetic lethal CRISPR-Cas9 screening. The screening showed that FSP1-targeting sgRNAs were greatly reduced in cells treated with RSL3, indicating that the combination of *FSP1* deletion and RSL3 treatment resulted in synthetic lethality. To further investigate whether ferroptosis activation by FSP1 inhibition exerts a novel antitumor effect, they generated a tumor xenograft mouse model using a GPX4 knockout, FSP1 knockout, and H460 large cell lung cancer cell line. Fer1 was then administered daily to inhibit ferroptosis. Washing out Fer1 did not change the survival rate of GPX4 knockout cells, whereas rapid death or synthetic lethality occurred in GPX4 knockout and FSP1 knockout cells. As U2OS and H460 cells are resistant to cystine depletion using an alternative pathway of glutathione production, this result emphasizes the need for an effective GPX4 inhibitor in vivo.

Doll et al. and Bersuker et al. discovered that the level of ferroptosis resistance among various cultured human cancer cell lines is correlated with intracellular FSP1 levels, suggesting that the modulation of the FSP1 level may have clinical significance. However, they pointed out that both FSP1 inducers and inhibitors are at the preclinical stage, and that GPX4 inhibitors like RSL3 have a low bioavailability.

NRF2 is an inhibitor of ferroptosis, and KEAP1-NRF2 is a key regulator of oxidative stress in cells. Activating mutations in KEAP1-NRF2 are frequently found in tumors of the lung, esophagus, and liver [67,68], and these mutations are associated with active tumor growth, resistance to anticancer drugs, and poor overall survival [69,70,71]. Although NRF2 is a major factor in tumorigenesis and chemotherapy resistance, there are currently no approved NRF2 inhibitors for treatment, with their use limited to mouse models [72]. Baird et al. [56] developed a new synthetic lethal assay using fluorescently labeled wild-type and Keap1 knockout cell lines to screen for compounds that selectively kill cells in an NRF2-dependent manner. As a result, they identified three compounds, namely, 17-dimethylaminoethylamino-17-demethoxygeldanamycin (17-DMAG), retaspimycin hydrochloride (IPI-504), and 17-allylamino-ethylamino-17-demethoxygeldanamycin (17-AAG), all of which exhibited synthetic lethality with NRF2 using geldanamycin as a scaffold. The product of the NRF2 target gene metabolizes quinone-containing geldanamycin compounds into a more potent HSP90 inhibitor, thereby increasing cytotoxicity. However, the synthetic lethal effect is limited to cells with an abnormal NRF2 activity. All three of these geldanamycin-derived compounds have been used in clinical trials [73,74,75,76,77,78,79] and are strong candidates for drugs that target NRF2 activity in currently untreatable cancers. However, the study does not state the type of cell death (ferroptosis or other) caused by synthetic lethality.

After screening various anticancer drugs and pathway-targeted anticancer drugs using wild-type and Keap1 knockout cells, Baird and Yamamoto identified several compounds showing increased toxicity against cells with a high Nrf2 activity [60]. Follow-up validation using eight human cancer cell lines revealed that mitomycin C, a DNA-damaging drug, was significantly more toxic to cells with an abnormal Nrf2 activity. Furthermore, as mitomycin C is already approved for clinical use [80,81], it is an appealing candidate for targeting NRF2 activation in human tumors that are currently untreatable.

Joly et al. [57] reported that the simultaneous targeting of GLUT1 and GSH synthesis may be a therapeutic approach to target tumors that survive in a glucose-dependent manner. Using a high xCT-expressing cell line and metabolomic analysis, they demonstrated that in cells undergoing cell death due to glucose deprivation, the levels of intracellular L-cysteine and its oxidized dimer L-cystine dramatically increased, resulting in their accumulation, whereas that of the antioxidant GSH was depleted.

Although ARID1A mutations are highly prevalent in ovarian follicular carcinoma (50%) and ovarian endometrial carcinoma (30%), as well as in many other types of cancer, none are a drug target [82,83,84]. ARID1A deficiency reduces xCT expression, causing an inadequate supply of cysteine, which is an important source of antioxidant GSH; therefore, it is specifically vulnerable to the inhibition of GSH and the catalytic subunit of glutamate-cysteine ligase synthase (GCLC), the rate-limiting enzyme in GSH synthesis. Ogiwara et al. [54] conducted a drug sensitivity screening using ARID1A wild-type and ARID1A knockout HCT 116 colon cancer cells to determine the selectivity of GSH metabolic factor inhibitors for ARIDIA-deficient cancers. The results showed that PRIMA-1 (APR-017), which covalently binds to the thiols of several polypeptides, and APR-246 (PRIMA-1 Met), a structural analog of PRIMA-1, were sensitive to ARIDIA-deficient cancers. APR-246 has been used in clinical trials for hematologic and prostate cancer [85]. Although the molecules and pathways involved in GSH synthesis, such as xCT and GSH/GCLC, are closely related to ferroptosis, they reported here that the inhibition of GSH/GCLC in ARID1A-deficient cancer cells caused apoptosis by ROS, but not ferroptosis [54]. Nevertheless, as it has been used in clinical trials and has a high potential for practical use, we included it in our review as a reported example of synthetic lethality.

To et al. [55] performed a genome-wide CRISPR screen using small-molecule mitochondrial inhibitors to identify the pathways that modulate mitochondrial dysfunction. GPX4 deletion enhances the toxicity of antimycin, oligomycin, ethidium bromide, and antimycin + oligomycin, all of which are small-molecule compounds that inhibit the mitochondrial complex. Furthermore, a synthetic lethal interaction between the loss of GPX4 activity and the inhibition of oxidative phosphorylation (OXPHOS) by oligomycin was confirmed in three cell lines (K562, HAP1, and HeLa). To rescue GPX4 knockout cells under oligomycin treatment, GPX4 was re-expressed using the standard selenocysteine insertion sequence. GPX4 has two major isoforms, namely, short GPX4 (sGPX4) and long GPX4 (lGPX4), which are localized in the cytoplasm and mitochondria, respectively. lGPX4 re-expression completely rescued the synthetic lethal interaction with oligomycin through the administration of the GPX4 inhibitors JKE-1674 or ML 210. These experiments support that mitochondrial GPX4 activity is sufficient to rescue synthetic lethal interactions and that mitochondria are an important site for lipid hydroperoxide accumulation and ferroptosis under OXPHOS inhibition. Furthermore, these results imply an “intramitochondrial” synthetic lethal interaction between GPX4 loss and oligomycin toxicity. The recent discovery of DHODH, which is a novel regulator of ferroptosis in the mitochondrial inner membrane, has also focused on the role of ferroptosis in mitochondria [29].

### 7.2. Related to Ferroptosis Inducers

Based on findings that oncogenic mutations alter the metabolic environment in tumor cells, Yusuf et al. [50] revealed a unique dependence on aldehyde dehydrogenase 3 family member A2 (Aldh3a2), which oxidizes long-chain aliphatic aldehydes, and vulnerability in different murine and human acute myeloid leukemia (AML) cells. By comparing normal primary mouse hematopoietic cells and their malignant counterparts in an ex vivo system that mimics the bone marrow microenvironment, and by performing a metabolism-limited genetic screen, they found that Aldh3a2 inhibition along with GPX4 inhibition leads to synthetic lethality in mouse and human AML cells. However, although GPX4 inhibition triggers ferroptosis, it has little effect on AML cells. In contrast, they found that leukemia cells, unlike normal bone marrow cells, prevent oxidative damage to cells in an Aldh3a2-dependent manner. Furthermore, aldehydes are the byproducts of increased OXPHOS and nucleotide synthesis in cancer, and are produced from lipid peroxides, which are the basis for ferroptosis. Thus, Aldh3a2 inhibition is a good example of a potential therapy taking advantage of the unique metabolic state of malignant cells, such as AML, and the benefits of synthetic lethality without affecting normal cells. However, there is no approved drug for Aldh3a2 inhibition, with efforts still in the preclinical stage.

Chen et al. [61,86] measured xCT expression by tissue microarray in CRC and found that xCT inhibition activates the transsulfuration pathway and maintains chemotherapy resistance. Specifically, RNA sequencing and in vitro functional assays showed that xCT blockade upregulates the expression of the transsulfuration pathway and that exogenous H_2_S partially counteracts chemotherapy resistance by increasing xCT stability. Additionally, aminooxy acetic acid and the xCT inhibitor erastin caused synthetic lethality in both colon cancer cell lines and xenograft mouse tumor models. Interestingly, they concluded that this synthetic lethality was mediated by increased ferroptosis and apoptosis.

Hereditary leiomyoma-renal cell carcinoma syndrome (HLRCC) is a hereditary cancer characterized by the inactivation of the Krebs cycle enzyme fumarate hydratase (FH). Kerins et al. [52] conducted drug sensitivity screening using the NCL-60 cancer cell line, focusing on FH inactivation to develop therapeutic agents for HLRCC, and identified drug sensitivity gene signatures according to their sensitivity to certain compounds. They also generated a FH knockout HK2-FH−/− UOK 262 cell line using the CRISPR/Cas9 system with sgRNA, and found that ferroptosis inducers (erastin, RSL3, ML162, and glutamate) showed synthetic lethality against FH knockout UOK 262 cells. Mechanistically, under FH−/− conditions, the accumulation of fumaric acid in cells causes posttranslational modification of C93 of GPX4, resulting in GPX4 dysfunction and increased susceptibility to ferroptosis.

Lorenzato et al. [58] focused on high-dose vitamin C therapy, which has a well-established safety profile in vivo, to overcome drug resistance to cetuximab, an EGFR-targeting antibody, in patients with advanced CRC. The concomitant administration of cetuximab and vitamin C to xenografts derived from patients with RAS/BRAF wild-type advanced CRC limited the growth of advanced CRC tissue and significantly delayed acquired resistance. Proteomics and metabolic flux analysis revealed that cetuximab inhibits glucose uptake and glycolysis, thereby interfering with glucose metabolism and promoting ROS production in a slow but gradual manner, whereas vitamin C disrupts iron homeostasis, increases ROS levels, and ultimately causes ferroptosis. In conclusion, the combination of vitamin C and cetuximab induces a synthetic lethal metabolic cell death program triggered by ATP depletion and oxidative stress that effectively suppresses the emergence of acquired resistance to anti-EGFR antibodies. Interestingly, vitamin C paradoxically produces ROS at pharmacological doses, despite being an antioxidant [86,87], and induces ferroptosis by exploiting this effect. Additionally, the advanced CRC cells used in this study were RAS wild-type, but it has been reported that vitamin C treatment causes cell death in advanced CRC cells with a RAS mutation [88,89,90].

Noting that cancer cells are often resistant to xCT inhibition by GSH deficiency, Okazaki et al. [53] conducted a synthetic lethal screening of a drug library to identify the agents that sensitize cancer cells resistant to GSH deficiency to the xCT inhibitor SAS, and identified dyclonine, an oral anesthetic that covalently inhibits ALDH. Furthermore, they found that the administration of dyclonine increased the levels of the cytotoxic molecule 4-hydroxynonenal in cells, and that this effect also occurred with SAS administration. Additionally, they determined that the ALDH3A1 expression was high in SAS-resistant head and neck squamous cell carcinoma (HNSCC) cells and that ALDH3A1 knockdown resulted in SAS sensitivity. The combination of dyclonine and SAS cooperatively inhibited the growth of HNSCC and gastric cancer, both of which highly express ALDH3A1 and are resistant to SAS monotherapy. These results provide a rationale for the application of dyclonine as a sensitizer for xCT-targeted cancer therapy. Interestingly, they reported that cell death induced by the combination of dyclonine and SAS resulted in non-programmed cell death.

HIF-2a, which is involved in the hypoxic response in tumors, is involved in colon cancer progression. Singhal et al. [62] created tamoxifen-inducible HIF-2a expressing murine intestinal tumors to identify HIF-2a dependence and vulnerability, and examined cell proliferation under anticancer drug treatment, including ferroptosis-inducing small molecules. They discovered that HIF-2a and the known ferroptosis activators, erastin, RSL3, and dimethyl fumarate (DMF), were synthetic lethal in colon cancer. DMF is FDA approved for treating multiple sclerosis and recurrent infections; the drug is a cell-permeable dimethyl ester of fumaric acid possessing immunomodulatory properties [91,92]. Tamoxifen-induced, intestine-specific deletion of Slc7a11 and overexpression of HIF-2a in mice showed that the colonic mucosa was histologically highly atypical, with high epithelial cell loss and high levels of 4HNE. Additionally, it was observed that the small intestine had a high iron concentration, making it more susceptible to ferroptosis.

Verma et al. [59] used synthetic lethal interaction-dependent drug combination high-throughput screening to identify drug combinations effective as targeted therapies for triple-negative breast cancer (TNBC). They reported that the coinhibition of bromodomain and extra-terminal (BET) and proteasomes induces ferroptosis and synthetic lethality in TNBC cell lines. Furthermore, using breast cancer patient-derived genomic and transcriptomic data from the METABRIC dataset and shRNA/CRISPR data from cell lines, the clinically promising synthetic lethal pairs in the screened drugs were ranked and the strength of each synthetic lethal interaction was estimated. The results of screening revealed that dual inhibition by BET with the bromodomain repeat sequences and specific terminal sequences and proteasomes was a promising choice. In addition, they identified two potential drug combinations that target members of the BET family. The first, targeting BET and CXCR2, was specific to mesenchymal subtypes of TNBC and induced apoptosis; the second, targeting BET and proteasomes, was effective against major TNBC subtypes and induced ferroptosis. Evidence of ferroptosis included an increase in intracellular iron and a decrease in glutathione level, as well as a decrease in GPX4 and key glutathione biosynthesis genes.

Thus far, we have introduced the literature on the relationship between ferroptosis and synthetic lethality parsed by the presence or absence of RAS mutations, but the relationship between RAS mutations and ferroptosis remains controversial. AML with RAS mutations is sensitive to chemotherapy, including cytarabine [93]. Yu et al. [51] focused on this fact and the presence of the ferroptosis-inducing agent erastin in RAS-mutated cells. They demonstrated that AML cells with the NRAS mutation Q61L underwent erastin dose-dependent mixed cell death associated with ferroptosis, apoptosis, necroptosis, and autophagy, with the joint induction of ferroptosis and necroptosis dominant. As the molecular mechanism underlying the joint induction of ferroptosis and necroptosis involved the activation of JNK and p38 signaling, it was concluded that the observed cell death was RAS-independent. It was also determined that cell death was not induced in several KRAS wild-type/mutant leukemia cell types, Jurkat (acute T-cell leukemia, RAS wild-type), THP-1 (NRAS G12D), K562 (chronic myeloid leukemia, RAS wild-type), or NB-4 (acute promyelocytic leukemia M3, KRAS A18D) cells. In addition to the increased antitumor activity of high concentrations of erastin as a single agent, they reported that low concentrations of erastin enhanced the sensitivity of HL-60 cells to chemotherapeutic agents (cytarabine/ara-C and doxorubicin/adriamycin), producing a synthetic lethal effect and helping overcome drug resistance in AML cells.

## 8. Challenges and Problems

Applying the concept of synthetic lethality to cancer therapy is very promising because it approaches mechanisms and genetic abnormalities that cannot be directly targeted by conventional therapeutic strategies (i.e., RAS mutations, hypoxia, and abnormalities in the metabolic environment). Furthermore, synthetic lethality targets the environment and conditions specific to malignant cells, and exhibits a low toxicity to normal cells [31,32,94]. However, more than 20 years after synthetic lethal strategies were proposed as targets for new anticancer drugs [95], their practical applications remain limited [30,96], in large part because genetic interactions in synthetic lethality are, by definition, lethal, making it difficult to recover and identify mutants, and because many synthetic lethality interactions are condition-dependent, and therefore are not always easy to reproduce [31].

Previous studies have described the efficacy of drugs already in clinical use, such as SAS [47], vitamin C, and mitomycin C, and drugs used in clinical trials like APR-246, as well as the efficacy of combinations of first-line drugs (cytarabine/ara-C and doxorubicin/adriamycin) and erastin for remission induction therapy in AML [49]. However, the efficacy of combinations of first-line drugs (cytarabine/ara-C and doxorubicin/adriamycin) with erastin in the induction of AML remission was also evaluated [49]; however, most of the substances described are at the preclinical stage. Erastin and its analogs have been investigated in several clinical trials, but the results have not been satisfactory [97,98]. There is also the issue that compounds like RSL3 have a low bioavailability [28].

Furthermore, as a mechanism of cell death, ferroptosis has only been recently reported, and much work remains to enable the targeting of these pathways for cancer treatment. Although some studies have reported that nonferroptotic cell death occurred with a ferroptosis inducer [47,53,54], others reported either an overlap of apoptosis and ferroptosis [61] or mixed cell death with ferroptosis, apoptosis, and autophagy [30]. These findings of cross-linkage between ferroptosis and other types of cell death need further clarification to effectively distinguish ferroptosis from other types of cell death in the future [49]. Ferroptosis can be described as a sensitive and context-dependent form of cell death, a definition that is neither simple to verify nor follow up. Morphological changes observed by electron microscopy, cell death induced by ferroptosis-inducing agents, inhibition of cell death by ferroptosis inhibitors, accumulation of lipid peroxides, and an increase in divalent iron ions are some of the methods used to demonstrate that cells are ferroptotic.

## 9. Prospects for Synthetic Lethal Strategies Using Ferroptosis

Ferroptosis is a type of cell death that is closely related to the cellular environment, such as oxidative stress and metabolic abnormalities, and is inherently compatible with synthetic lethal strategies. The advantage of a synthetic lethal strategy is that new drug effects are expected when combined with existing approved drugs. In addition to SAS, vitamin C, mitomycin C, and DMF, all of which are currently approved drugs for use in vivo, other drugs such as geldanamycin-derived compounds (17-DMAG, IPI-504, and 17-AAG) and APR-246 are still at the clinical trial stage. There are also expectations for the clinical application of erastin and its analogs, whose efficacy has yet to be confirmed in clinical trials. A novel compound consisting of a tumor-targeting molecule bound to an erastin analog dramatically increased cell death via ferroptosis in a pancreatic cancer model; it was markedly more potent than erastin at inducing ROS production and ferroptosis [99]. Additionally, erastin analogs developed by introducing reactive carbonyls were found to greatly improve the potency, solubility, and metabolic stability of erastin compounds [100]. Future research is expected to further elucidate the drugs and their combinations that can be effectively used in vivo.

## Figures and Tables

**Table 1:** The characteristics of ferroptosis and other type of RCD (modified from 4, 11, 12).

	Ferroptosis		Apoptosis	Necroptosis	Autophagy
Morphological features	Smaller mitochondria, reduced mitochondria crista, elevated mitochondrial membrane densities, increased rupture of mitochondrial membrane		Cell rounding, nuclear condensation, membrane blebbing, apoptotic body formation	Cell swelling, rupture of plasma membrane, moderate chromatin condensation	Formation of double membraned autolysosomes (Autophagic vacuolization)
Biological features	Iron accumulation and lipid peroxidation		Activation of caspases and DNA fragmentation	Drop in ATP levels, cytosolic necrosome formation	increased autophagic flux and lysosomal activity
Regulatory pathways	Xc- /GPX4, MVA, sulfur transfer pathway, P62-Keap1-NRF2 pathway, P53/SLC7A11, ATG5-ATG7-NCOA4 pathway, P53-SAT1-ALOX15 pathway, HSPB1-TRF1, FSP1-COQ10-NAD(P)H pathway		Death receptor pathway, mitochondrial pathway and endoplasmic reticulum pathway; Caspase, P53, Bcl-2 mediated signaling pathway	TNF-R1 and RIP1/RIP3-MLKL related signaling pathways; PKC-MAPK-AP-1 related signaling pathway; ROS-related metabolic regulation pathway	mTOR, Beclin-1, P53 signaling pathway
Major regulators	Positive	TFR1, ACSL4, NCOA4, VDAC2/3	Caspase, pro-apoptotic BCL2 family (e.g., BAX), TP53	RIPK1, RIPK3, MLKL	ATG5, ATG7, Becilin-1, Other ATG famiy proteins
	Negative	GPX4, SLC7A11, FSP1 NRF2(NFE2L2), HSPB1, HSPA5	anti-apoptotic BCL2 family (e.g., BCL2)	ESCRT-III, cIAPs, LUBAC, PPM1B, and AURKA	mTOR
	Dual	TP53			

ACSL4 acyl-CoA synthetase long-chain family member 4, ALOX-15 arachidonate lipoxygenase 15, AP-1 activator protein-1, ATG5 autophagy-related 5, ATG7 autophagy-related 7, AURKA Aurora Kinase A, BAX BCL-2 associated protein X, BCL-2 B-cell lymphoma 2, cIAPs cellular inhibitor of apoptosis proteins, COQ10 coenzyme Q10, ESCRT endosomal sorting complexes required for transport, FSP1 ferroptosis suppressor protein 1, GPX4 glutathione peroxidase 4, HSPB1 heat shock protein beta-1, Keap1 Keleh-like ECH-associated protein 1, LUBAC linear ubiquitin chain assembly complex, MAPK mitogen-activated protein kinase, MLKL mixed lineage kinase domain like protein, mTOR mammalian target of rapamycin, MVA mevalonate, NCOA4 nuclear receptor coactivator 4, NRF2 nuclear factor erythroid 2-related factor 2, PKC protein kinase C, PPM1B Protein Phosphatase, Mg^2+^/Mn^2+^ Dependent 1B, RIP receptor-interacting serine/threonine kinase, ROS reactive oxygen species, SAT1 spermidine/spermine N1-acetyltransferase 1, SLC7A11 solute carrier family 7 member 11, system Xc- cysteine/glutamate transporter receptor, TFR1 transferrin receptor 1, TNF-R1 tumor necrosis factor R1.

**Table 2:** The common inducers and inhibitors of ferroptosis.

Inducer	Class1: Inhibit system Xc- and prevent cystine import	Erastin, Sorafenib, Sulfasalazine
	Class2: Inhibit GPX4	RSL3, (1S,3R)-RSL3, DPI7, DPI10
	Class3: Degrade GPX4, bind to SQS, and deplete antioxidant CoQ10	FIN56
	Class4: Oxidize ferrous iron and lipidome directly, and inactivate GPX4 indirectly	FINO2
	Supplement: Target VDACs, degrade GPX4	Erastin
Inhibitor	Class1: Inhibit accumulation of iron	DFO, Deferoxamine mesylate, 2,2′-pyridine
	Class2: Inhibit lipid peroxidation	Fer-1, SRS11–9, SRS16–86, Liproxststatin-1, Vitamin E

Ac—acetaminophen; ART—artesunate; COQ1—coenzyme Q10; DFO—deferoxamine; Fer-1—Ferrostatin-1; GPX4—glutathione peroxidase 4; GSH—glutathione; RSL3—Ras-selective lethal small molecule 3; VDACs—voltage-dependent anion channels.

**Table 3:** Synthetic Lethality and Regulators of Ferroptosis.

Author	Synthetic Lethal Factors (A)	Synthetic Lethal Factors (B)	RAS Mutation	Summary of Synthetic Lethality	Tissues and Cells	Verification of Ferroptosis							Other Type of Cell Death
						Increase in Intracellular Reactive Oxygen Species and Lipid Peroxides	Intracellular Iron Accumulation/Inhibition by Iron Chelators	Cell Death by Ferotosis Inducer	Morphological Changes Characteristic of Ferroptosis	Bioenergetic Changes: Intracellular ATP Depletion	Lack of Other PCD Features	Escape of Cell Death by Ferroptosis Inhibitors	
Yang, W.S., et al. [13]	RSL3, ML162, anf DPI10		oncogenic-HRAS	RSL3, ML162, and DPI10 induce ferroptosis in engineered oncogenic-RAS fibroblast-derived tumorigenic cell lines	oncogenic-HRAS cells, 117 human cancer cell lines	○		○			○		
Yu, Y., et al. [51]	Erastin	Cytarabine and Doxorubicin		Erastin induces cell death in AML cells in a dose-dependent manner through a mixture of ferroptosis, apoptosis, necroptosis, and autophagy.	HL-60 cells (AML, NRAS_Q61L)								mixed types of cell death associated with ferroptosis, apoptosis, necroptosis, and autophagy
Chio, I.I.C., et al. [46]	MK 2206 (pan-AKT inhibitor)	BSO	oncogenic-KRAS	Combined targeting of AKT and glutathione synthesis inhibits pancreatic cancer	human and mouse Kras mutant PDA cells, Suit2 xenograft model							○	
Kerins, M.J., et al. [52]	Erastin, RSL3, ML162, glutamate	FH deficiency		Ferroptosis inducers are selectively toxic to FH−/− cell line UOK262, because C93 of GPX4 is post-translationally modified by fumarates that accumulate in conditions of FH−/−, and C93 modification represses GPX4 activity.	HK2 fumarate hydratase knockout cell lines	○		○				○	
Okazaki, S., et al. [53]	Sulfasalazine (xc- inhibitor)	Dyclonine (oral anesthetics)		Sulfasalazine-resistant head and neck squamous cell carcinoma (HNSCC) cells highly express ALDH3A1 and knockdown of ALDH3A1 sensitized these cells to sulfasalazine. The combination of dyclonine and sulfasalazine cooperatively suppressed the growth of highly ALDH3A1-expressing HNSCC or gastric tumors that were resistant to sulfasalazine monotherapy.	Sulfasalazine-resistant human HNSCC cells								non-programmed cell death
Bersuker, K., et al. [28]	RSL3	FSP1		In CRISPR-Cas9 screening, RSL3 induces synthetic lethality in U-2 OS FSP1 knockout cell lines. FSP1 acts parallel to GPX4 to inhibit ferroptosis.	U-2 OS FSP1 knockout cell lines			○					
Ogiwara, H., et al. [54]	GCL catalytic subunit (GCLC)	ARID1A deficiency		Cancer cells lacking ARID1A are specifically vulnerable to glutathione and inhibition of GCLC.	ARID1A-knockout HCT116 colon cancer cells								apoptosis
To, T.L., et al. [55]	GPX4	Mitochondrial Inhibitors (antimycin, oligomycin, ethidium bromide)		Genome-wide CRISPR screening using small molecule mitochondrial inhibitors showed that genes involved in the glycolytic system (PFKP), pentose phosphate pathway (G6PD), and defense against lipid peroxidation (GPX4) were closely associated with synthetic lethality.	K562, HAP1, HeLa	○					○		
Hu, K., et al. [22]	SLC7A10		oncogenic-KRAS	In KRAS-mutant lung adenocarcinoma transplanted mice, treatment with SLC7A11 inhibitor (HG106) resulted in tumor suppression and prolonged survival.	KRAS-mutant lung adenocarcinoma cells, preclinical lung cancer mouse model								apoptosis
Kwon, O.S., et al. [47]	Erastin		oncogenic-RAS	Erastin is a synthetic lethal compound against cancer expressing an oncogenic RAS. The activity of transcription factors, including NRF2 and AhR, serve as important markers of erastin resistance.	mesenchymal lung cancer cell lines			○	○		○		
Sugiyama, A., et al. [49]	Sulfasalazine (xc- inhibitor)		JNK (RAS effector) activation	Sulfasalazine is highly cytotoxic in paclitaxel-resistant uterine serous carcinoma. Interaction of ROS accumulation and JNK pathway activation increases susceptibility to SAS-induced ferroptosis.	human uterine serous carcinoma cell lines			○				○	
Yusuf, R.Z., et al. [50]	RSL3, GPX4 knock down	Aldh3a2 inhibition		Aldh3a2 inhibition in combination with GPX4 inhibition leads to synthetic lethality in mouse and human AML cells.	human AML cells, Aldh-mut and Aldh-Ctrl mice were used to generate MLL-AF9 leukemia through retroviral transduction	○					○	○	
Baird, L., et al. [56]	NRF2	17-AAG, 17-DMAG, IPI-504		17-AAG is synthetic lethal with NRF2 in human cancer cell lines	Keap1 knockout cells	No description of what type of cell death.							
Joly, J. H., et al. [57]	GSH	GLUT1		Co-targeting GLUT1 and GSH synthesis induces synthetic lethal cell death in high xCT-expressing cell lines susceptible to glucose deprivation	xCT-high cell lines of glioblastoma and Ewing’s sarcoma	No description of what type of cell death.							
Lorenzato, A., et al. [58]	vitamine C	Cetuximab		The combination of vitamin C and cetuximab causes synthetic lethality triggered by ATP depletion and oxidative stress. This in turn suppresses the acquisition of resistance to anti-EGFR antibodies.	Advanced colorectal cancer patient-derived xenografts	○	○		○			○	
Verma, N., et al. [59]	Ferroptosis inducers (FIN56, erastin)	BET inhibitor (JQ1) + proteasome inhibitor (BTZ)		Co-inhibition of BET (bromodomain and extra-terminal) and proteasome induces ferotosis and synthetic lethality in triple-negative breast cancer (TNBC) cell lines.	TNBC cell lines	○	○						
Baird, L. and M. Yamamoto. [60]	NRF2	Mitomycin C		Aberrant NRF2 activation confers enhanced mitomycin C sensitivity in human cancer cell lines	NRF2-activated human cancer cells	No description of what type of cell death.							
Chen, S., et al. [61]	erastin	AOAA		AOAA and Erastin resulted in synthetic lethality both in vitro and in vivo, which was mediated through increased ferroptosis and apoptosis.	Tissue microarrays of colorectal cancer								apoptosis + ferroptosis
Singhal, R., et al. [62]	Erastin, RSL3, DMF	HIF-2α		Ferroptosis inducer (Erastin, RSL3) and DMF led to selective synthetic lethality in HIF-2a expressing tumor enteroids.	colon cancer cells and colon tumors in mice	○	○	○			○		

ATP, Adenosine tri-phosphate; PCD, Programmed cell death; AML, acute myeloid leukemia; BSO, L-Buthionine-(S,R)-Sulfoximine; PDA, Pancreatic ductal carcinoma; GCL, Glutamate cysteine ligase; MLL, Mixed lineage leukemia; BET, bromodomain and extra-terminal; BTZ, bortezomib; AOAA, Aminooxyacetic acid; DMF, N,N-dimethylformamide; HIF-2α, hypoxia-inducible factor 2α. ○, applicable; blank, not applicable.

## Data Availability

No data were used in this review.
